# Hexokinases link DJ-1 to the PINK1/parkin pathway

**DOI:** 10.1186/s13024-017-0212-x

**Published:** 2017-09-29

**Authors:** David N. Hauser, Adamantios Mamais, Melissa M. Conti, Christopher T. Primiani, Ravindran Kumaran, Allissa A. Dillman, Rebekah G. Langston, Alexandra Beilina, Joseph H. Garcia, Alberto Diaz-Ruiz, Michel Bernier, Fabienne C. Fiesel, Xu Hou, Wolfdieter Springer, Yan Li, Rafael de Cabo, Mark R. Cookson

**Affiliations:** 1 Cell Biology and Gene Expression Section, Laboratory of Neurogenetics, National Institute on Aging, National Institutes of Health, Building 35, Room 1A116, 35 Convent Drive, MSC 3707, Bethesda, MD 20892-3707 USA; 2 Experimental Gerontology Section, Translational Gerontology Branch, National Institute on Aging, National Institutes of Health, Baltimore, MD USA; 3 Department of Neuroscience, Mayo Clinic, Jacksonville, FL USA; 4 Mayo Clinic Graduate School of Biomedical Sciences, Jacksonville, FL USA; 5 Protein/Peptide Sequencing Facility, National Institute of Neurological Disorders and Stroke, National Institutes of Health, Bethesda, MD USA

**Keywords:** Proteomics, RNA-Seq, Metabolomics, Systems biology, Parkinson’s disease, Mitophagy, Parkin, PINK1, DJ-1, AKT, Hexokinase

## Abstract

**Background:**

Early onset Parkinson’s disease is caused by variants in *PINK1*, *parkin*, and *DJ-1*. PINK1 and parkin operate in pathways that preserve mitochondrial integrity, but the function of DJ-1 and how it relates to PINK1 and parkin is poorly understood.

**Methods:**

A series of unbiased high-content screens were used to analyze changes at the protein, RNA, and metabolite level in rodent brains lacking DJ-1. Results were validated using targeted approaches, and cellular assays were performed to probe the mechanisms involved.

**Results:**

We find that in both rat and mouse brains, DJ-1 knockout results in an age-dependent accumulation of hexokinase 1 in the cytosol, away from its usual location at the mitochondria, with subsequent activation of the polyol pathway of glucose metabolism in vivo. Both in the brain and in cultured cells, DJ-1 deficiency is associated with accumulation of the phosphatase PTEN that antagonizes the kinase AKT. In cells, addition of an inhibitor of AKT (MK2206) or addition of a peptide to dissociate association of hexokinases from mitochondria both inhibit the PINK1/parkin pathway, which works to maintain mitochondrial integrity.

**Conclusion:**

Hexokinases are an important link between three major genetic causes of early onset Parkinson’s disease. Because aging is associated with deregulated nutrient sensing, these results help explain why DJ-1 is associated with age-dependent disease.

**Electronic supplementary material:**

The online version of this article (10.1186/s13024-017-0212-x) contains supplementary material, which is available to authorized users.

## Background

Research into the genetic basis of Parkinson’s disease (PD) has identified many mutations that cause monogenic PD as well as variants that increase the risk of developing sporadic PD [1]. One of the major strategies to identify genetic mechanisms of PD risk has been the application of unbiased surveys of the genome, such as exome sequencing and genome-wide association studies. In the past decade, tools for analysis of the transcriptome, proteome, and metabolome have been refined enough to begin to approach the utility of those used for genomics. Therefore, it has been argued that applying unbiased high-information content approaches, or ‘omics’, would have a similarly accelerating effect on biological understanding of the functions of genes associated with PD [2].

One specific gene that causes a recessive early onset form of PD is *Park7,* which encodes the protein DJ-1 [3]. All known mutations in DJ-1 appear to act as loss of function variants [4], suggesting that identifying the normal DJ-1 function is critical to understanding its role in disease. However, the normal function of DJ-1 is not yet resolved. Prior to being designated as a gene for early onset Parkinson’s disease, DJ-1 had been reported to have weak oncogenic properties via binding to the small GTPase Ras [5], and was then associated with male fertility in rats [6]. DJ-1 protein is responsive to oxidative stress [7–9], with cysteine-106 identified as the most sensitive to oxidative modification through formation of a cysteine-sulfinic acid adduct [7–9]. Several cellular functions have been attributed to DJ-1, including a transcriptional co-activator [10], a negative regulator of the PI3K/AKT signaling cascade through inhibition of phosphatase and tensin homologue (PTEN) [11–13], a chaperone for alpha synuclein [14], an RNA binding protein [15], and a copper and mercury chaperone [16, 17]. DJ-1 has also been claimed to be an enzyme with peroxidase [18], protease [19], glyoxalase [20] and deglycase activities [21]. The study of DJ-1 function in vivo has been hampered by the fact that none of these proposed activities have been conclusively shown to be physiologically relevant, and some such as the deglycase activity may be experimental artifacts [22].

Variants in *PINK1* and *PARK2* (parkin) cause early onset Parkinson’s disease in humans with a similar clinical presentation as *DJ-1* mutations [23, 24]. PINK1 and parkin have been implicated in mitophagy, a selective degradative pathway that limits accumulation of damaged mitochondria [25]. Mutations in *FBXO7* are responsible for a form of atypical juvenile onset PD, and FBXO7 has been shown to help mediate the PINK1/parkin pathway [26, 27]. DJ-1 does not appear to be directly involved in mitophagy, but does protect cells from mitochondrial dysfunction, likely due to its involvement in oxidative stress responses [28, 29]. In *Drosophila melanogaster* models, DJ-1 deletion partially phenocopy parkin mutants but only in aged animals [28]. Thus, DJ-1 appears to act parallel to the PINK1/parkin pathway but the precise relationship between these three genes remains obscure.

Here, the effects of DJ-1 knockout in the brain of rat and mouse models were determined using a series of unbiased high-content methods. A salient feature of DJ-1 deficiency in rodent brain tissue was the relocalization of hexokinase 1 (HK1) from the outer mitochondrial membrane to the cytosol, and an associated buildup of products of the polyol pathway of glucose metabolism. We find accumulation of the phosphatase PTEN occurs in knockout animals and in cultured cells, suggesting that this is an immediate downstream consequence of DJ-1 deficiency. Additional experiments in cultured cells also show that either pharmacological inhibition of AKT or dissociation of hexokinase from mitochondria led to the inhibition of the PINK1/parkin pathway. These results indicate that constitutive loss-of-function mutations in DJ-1 are associated with the deregulation of nutrient sensing and mitochondrial dysfunction, two central hallmarks of aging and age-dependent phenotypes of disease in humans [30].

## Methods

### DJ-1 knockout rats and mice

Frozen brains from 6-month-old male DJ-1 knockout rats and wild type (WT) controls on a Long Evans background were a gift from the Michael J. Fox Foundation. The brains were stored as frozen hemibrains (cerebrum, cerebellum, and brainstem) until use. DJ-1 knockout mice [31] were a gift from Dr. Huaibin Cai and were additionally backcrossed for 7 generations with C57BL/6 mice prior to use in experiments. The mice were given access to food and water ad libitum and housed in a facility with 12-h light/dark cycles. DJ-1 heterozygous breeding pairs were used to generate littermate animals with WT and homozygous knockout genotypes that were used in these experiments. The mice were sacrificed at the indicated age and the brains were rapidly removed on ice. The cerebellum was removed and the brains were bisected along the longitudinal fissure.

### Subcellular fractionation of rodent brains

The frozen rat hemibrains were thawed and homogenized in 10 mL ice-cold mitochondrial isolation buffer (5 mM HEPES pH 7.3, 0.225 M mannitol, 0.05 M sucrose) and a 0.5 mL aliquot was taken and immediately frozen as the whole brain homogenate. Mouse brains were homogenized in 1 mL isolation buffer and 0.05 mL was saved as whole brain homogenate. The remaining homogenate was centrifuged at 1200*×g* for 3 min at 4 °C. The resulting supernatant was saved and the pellet was resuspended in isolation buffer then centrifuged again at the same settings. The two S1200 fractions were combined then centrifuged (12,000*×g*, 10 min, 4 °C). The S12,000 was further centrifuged (100,000*×g*, 1 h, 4 °C) and the supernatant was immediately frozen as the cytosol-enriched fraction. The 12,000×*g* pellet from the above step was resuspended in isolation buffer containing 0.002% digitonin to rupture synaptosomes and centrifuged again (12,000×*g*, 10 min, 4 °C). The pellet was washed by resuspension in isolation buffer and then centrifuged once more (12,000*×g*, 10 min, 4 °C). The resulting pellets were immediately frozen as mitochondria-enriched fractions. Frozen mitochondria-enriched fractions were lysed by resuspension in iTRAQ lysis buffer (0.3 M HEPES pH 8.0, 2% *w*/*v* CHAPS, and 1 mM EDTA) and left for 15 min on ice before centrifugation (16,000*×g*, 15 min, 4 °C) to clear the lysate of insoluble material.

### Proteomic analysis of rat and mouse brains

Protein concentrations of mitochondria-enriched and cytosolic fractions from the rat and mouse brains were determined using a 660 nm protein assay (Pierce). Equal amounts of protein from each WT rat or mouse fraction were pooled to created standards for the rat mitochondria-enriched, rat cytosolic, and mouse cytosolic fractions. The standards and each individual sample were labeled with iTRAQ 8-plex reagents as previously reported [32]. Six 2D–LC-MS/MS runs were performed to analyze the samples (2 runs with all rat cytosolic fractions, 2 runs with all rat mitochondria-enriched fractions, and 2 runs with the mouse cytosolic fractions). The respective pooled standard was used as a reference during each run. 2D–LC-MS/MS was performed as we have previously reported [32].

Mascot (v2.4) [33] was used to identify peptides from each run by searching against either the Swissprot Rat or Mouse database. The quantitation method was iTRAQ 8plex, peptide mass tolerance was ±20 ppm, fragment mass tolerance was ±0.3 Da, maximum number of missed cleavages was 1–2, fixed modifications were iTRAQ8plex (N-term) and iTRAQ8plex (K), and variable modifications were Methylthio (C), Oxidation (M), and iTRAQ8plex (Y). The intensity of each iTRAQ label was divided by the intensity of the pooled standard’s label in channel 113 to obtain a ratio for each channel. Protein ratios were calculated as weighted peptide ratios from a minimum of 2 unique peptides. Peptide ratios were normalized so that the median ratio was set to 1 and outlier peptide ratios were removed using Mascot’s automatic removal feature.

Only proteins that had quantification in each iTRAQ channel in both runs were used for statistical analysis. The ratios for each sample were log_2_ transformed and then the two groups were compared using Welch’s t-tests. *P* values were adjusted using the Benjamini-Hochberg procedure to control for the false discovery rate (FDR).

### RNA-sequencing of rat brain mRNA

Frozen rat hemibrains were homogenized in 7 mL ice-cold Trizol reagent (Invitrogen) using glass Dounce homogenizers and RNA was purified according to the standard Trizol reagent protocol. RNA was quantified using a NanoDrop spectrophotometer and RNA integrity was measured using RNA Nano Chips and an Agilent 2100 Bioanalyzer. cDNA libraries were generated from purified mRNA using TruSeq RNA Sample Preparation Kits (v2, Illumina) according to the manufacturer’s instructions. The cDNA from each hemibrain was given a unique DNA barcode then samples were pooled and sequenced using an Illumina HiSeq 2000.

The standard Illumina pipeline was used to generate fastq files, which were aligned to the rat genome (rn5) using Tophat [34] and Bowtie [35]. The Ensembl gene transfer format (gtf) file Rattus_norvegicus.Rnor_5.0.73.gtf was used to build Bowtie indices. The Python framework HTSeq [36] was used to annotate and quantify reads. For gene expression analysis, we used the DESeq2 package [37]. Prior to calculating test statistics for each gene, we filtered out the lowest 40% of genes based on their mean counts. The counts for the resulting genes were then normalized using a variance-stabilizing transformation and the two groups were compared using a negative binomial test. *P* values were adjusted using the Benjamini-Hochberg method.

The list of genes with significantly altered expression (Benjamini-Hochberg adjusted *p* < 0.05) was analyzed using WebGestalt [38]. Of the 2080 gene IDs uploaded to the program, 1611 mapped to unique Entrez Gene IDs and were used in the analysis while 464 could not be mapped and were excluded. The reference set was rnorvergicus_genome, statistical method was hypergeometric, and multiple tests adjustment was Benjamini Hochberg. We ran a KEGG enrichment analysis and also a transcription factor target enrichment analysis. The latter tests for enrichment of genes with promoter regions +/− 2 kb from specific transcription factor binding sites. We report the top ten groups returned by each analysis, and also searched the entire list of enriched transcription factor targets with Benjamini-Hochberg adjusted *p* values <0.05 for AKT substrates listed in an online database (http://www.cellsignal.com/common/content/content.jsp?id=science-tables-akt-substrate).

### Western blots

Proteins were separated using 18 well 4–20% Criterion TGX gels (Bio-Rad) and transferred to PVDF membranes using a Trans-Blot Turbo system (Bio-Rad). Western blotting was performed according to the protocol set forth by LI-COR for Near-Infrared Western Blot Detection. Incubations with primary antibodies were done overnight at 4 °C, while those with secondary antibodies were done for 1 h at RT. Blots were imaged using an Odyssey CLx system and quantification was done using instrument’s Image Studio software. The antibodies used were β-actin (Sigma A1978, 1:40,000–200,000), DJ-1 (Abcam ab76008, 1:4000–10,000), CRYAB (Abcam ab13496, 1:1000), HSP60 (Abcam ab46798, 1:10,000), GLUL (Millipore MAB302, 1:40,000), GAD2 (Cell Signaling 5843S, 1:4000), PHGDH (Atlas Antibodies HPA021241, 1:4000), NEFL (Cell Signaling 2835S, 1:4000), HK1 (Cell Signaling 2024S, 1:4000), pan AKT (Cell Signaling 2920S, 1:1000), pAKT S473 (Cell Signaling 4060S, 1:1000), pAKT T308 (Cell Signaling 13,038, 1:1000), GSK3β (Cell Signaling 9315, 1:1000), pGSK3β S9 (Cell Signaling 5558, 1:1000), HK2 (Cell Signaling 2867S, 1:2000), VDAC (Abcam ab14734, 1:2000), parkin (Cell Signaling 4211, 1:2000), PTEN (Cell Signaling 9556, 1:1000), and pPTEN S380 (Cell Signaling 9551, 1:1000).

### Immunohistochemistry

For immunohistochemistry, we used coronal sections from the brains of one year-old mice that we have previously described [39]. All steps were done on using free-floating sections on a shaker. First, the sections were quenched with glycine (0.3 M in PBS) for 20 min and then washed in PBS three times for 5 min each at RT. Sections were blocked for 1 h at RT in blocking buffer (PBS supplemented with 1% *w*/*v* BSA, 0.3% Triton X-100, and 10% *v*/v donkey serum), which was also used to dilute primary and secondary antibodies in later steps. Sections were washed again in PBS three times for 5 min before being incubated overnight in the any combination of three separate species antibodies at one time: HK1 (LS-B6098, mouse monoclonal, 1:1000), TH (PelFreez P40101, rabbit polyclonal, 1:2000), GFAP (Abcam ab53554, goat polyclonal, 1:1000), TOM20 (Santa Cruz sc-11,415, rabbit polyclonal, 1:1000), and Orexin-A (Santa Cruz sc-8070, goat polyclonal, 1:1000) at 4 °C. The following day, the sections were left at room temperature in primary antibody for 1 h then rinsed in PBS three times for 5 min each. Three secondary antibodies were incubated with the sections (Alexa Fluor 488 donkey anti-mouse IgG, 1:500; Alexa Fluor 568 donkey anti-goat IgG, 1:500; Alexa Fluor 647 donkey anti-rabbit IgG, 1:500) for 1 h at RT and protected from light. Sections were then incubated with Hoechst 33,342 in PBS (1:10,000 dilution) for 15 min. Finally, the slices were washed three times for 5 min each in PBS, mounted on glass slides, placed in a dryer at 37 °C for 15 min, and coverslipped using Prolong Gold mounting media. The slides were cured at RT for at least 24 h before being imaged on a Zeiss 780 confocal microscope.

### Immunocytochemistry

Cells were grown on glass coverslips coated with poly-D-lysine and laminin and fixed with ice-cold 4% paraformaldehyde in PBS for 15 min. Next, cells were permeabilized with 0.1% *v*/v Triton X-100 in PBS for 10 min, and then blocked with blocking buffer (10% FBS in PBS with 0.1% v/v Triton X-100) for 1 h. Blocking buffer was also used to dilute primary and secondary antibodies. The primary antibodies used were HK1 (LS-B6098, mouse monoclonal, 1:1000), HK1 (Abcam ab150423, rabbit monoclonal, 1:1000), GFAP (Abcam ab53554, goat polyclonal, 1:1000), and were incubated with the cells for 1 h at RT or overnight at 4 °C. The secondary antibodies (Alexa Fluor 488 donkey anti-mouse IgG, 1:500; Alexa Fluor 568 donkey anti-rabbit IgG, 1:500; Alexa Fluor 647 donkey anti-goat IgG, 1:500) were incubated with cells for 1 h at RT.

### Metabolomics

Frozen cerebral hemispheres from 4-month-old mice were sent to Metabolon Inc. where they were processed and analyzed for 240 metabolites using a combination of GC/MS and LC/MS. Values for any sample missing data were imputed as the minimum detectable amount of the metabolite in question. The raw spectral intensities for each metabolite were subsequently rescaled so that the median of all samples was equal to 1. This data was then log_2_ transformed and the genotypes were compared using Welch’s t-test. *P* values were then adjusted using the Benjamini-Hochberg method, and an adjusted *p* value cutoff of 0.05 was used to determine significance.

### Primary astrocyte experiments

Astrocyte-enriched primary cultures were prepared from the cerebra of mouse pups between postnatal days 1–3 and cultured in DMEM +10% FBS. At 8 days in vitro (DIV), flasks were shaken at 300 rpm for 3 h and the media was removed to eliminate microglia. These cultures were not tested for the presence of mycoplasma. For IGF-1 stimulation, the cells were plated in 6 well plates on 20 DIV. On 25 DIV, the cells were serum-starved for 3 h and then treated with 10 nM IGF-1 (Sigma 13,769). After five minutes, the plates were washed twice with PBS and then frozen with liquid nitrogen. The cells were lysed in the wells using RIPA buffer (50 mM Tris HCl pH 7.5, 150 mM NaCl, 1% Triton X-100, 0.5% sodium deoxycholate, 0.1% SDS) with protease and phosphatase inhibitor cocktails (Sigma P8340, P5726, P0044, 93,482, and T1952). For transfection experiments, DJ-1 knockout astrocytes were transfected using pDEST40 LacZ, wildtype DJ-1, or C106A DJ-1 plasmids [40]. The cells were serum starved for 3 h, stimulated with 10 nM IGF-1 for 5 min, then fixed and immunocytochemistry was performed as outlined above. The primary antibodies were V5 (Abcam ab9113, chicken polyclonal, 1:500), pAKT S473 (Cell Signaling 4060, rabbit monoclonal, 1:500), and AKT (Cell Signaling 2920, mouse monoclonal, 1:500).

### HeLa cell experiments

HeLa cells stably expressing YFP-parkin and MitoDsRed2 have been previously described [41]. Cells were seeded in 96-well plates at a density of 5000 cells per well. The cells were incubated with MK2206 (10 μM) for 24 h prior to a one-hour treatment with either DMSO or 5 μM CCCP or 2 h with 10 μM valinomycin. The hexokinase II VDAC-Binding Domain Peptide (Millipore 376,816) was resuspended in water at 1 mg/mL (250 μM) and added to cells to reach a final concentration of 100 μM. An equivalent amount of water was added to the control wells, and the cells were incubated for 30 min prior to a one-hour treatment with 5 μM CCCP or DMSO. Plates were washed once with PBS and then fixed with 4% paraformaldehyde in PBS containing 1 μg/mL Hoechst. Immunocytochemistry using a rabbit polyclonal antibody raised against pS65-ubiquitin (1:1000) [42] was performed as described above (Alexa Fluor 647 donkey anti-rabbit IgG, 1:500). Plates were scanned using a Cellomics VTI Arrayscan and at least 400 cells per well were used for quantification with the Cellomics software.

Cells were seeded in 10 cm dishes for immunoprecipitation experiments and incubated with either MK2206 (10 μM) or DMSO for 24 h prior to treatment with 10 μM CCCP for one hour. The cells were lysed using lysis buffer (100 mM HEPES pH 7.7, 250 mM KCl, 2 mM MgCl_2_, 2 mM EDTA, 10% glycerol, 1.0% digitonin, 7 mM β-mercaptoethanol, 1× protease inhibitor (Roche), and 1× phosphatase inhibitor (Pierce). Lysates were precleared with nAb agarose control beads (Allele Biotech) prior to immunoprecipitation using GFP nAb agarose beads (Allele Biotech).

### Statistics

Statistical analyses were performed in R (https://www.R-project.org/) and Prism (Graphpad Software). For iTRAQ studies, samples sizes were chosen to be able to detect ~25% change (α = 0.00004, β = 0.8) based on prior experiments [32]. For RNA-Seq, metabolomics, and Western blot experiments no statistical method was used to determine sample size. No randomization was used for animal studies. Investigators were blinded to the genotype of the animals for immunohistochemistry experiments examining colocalization of HK1 and TOM20. Where indicated, *t*-tests used the Welch’s correction to account for the possibility of unequal variances. Normal distributions were assumed based on visual interpretation of the data. Outliers were detected in the primary astrocyte pAKT immunocytochemistry experiment using the Tukey method and were omitted prior to statistical tests.

## Results

### Cytosolic HK1 increases with age in DJ-1 knockout rodent brains

In order to understand the effects of loss of DJ-1 function in vivo, we applied isobaric tags for relative and absolute quantitation (iTRAQ) proteomics to analyze protein abundance in mitochondrial and cytosol enriched fractions from the brains of WT and DJ-1 knockout rodent models. We first used a cohort of 6-month-old DJ-1 knockout rats, which have been reported to have progressive neurodegeneration and behavioral deficits reminiscent of PD [43].

**Fig. 1:**
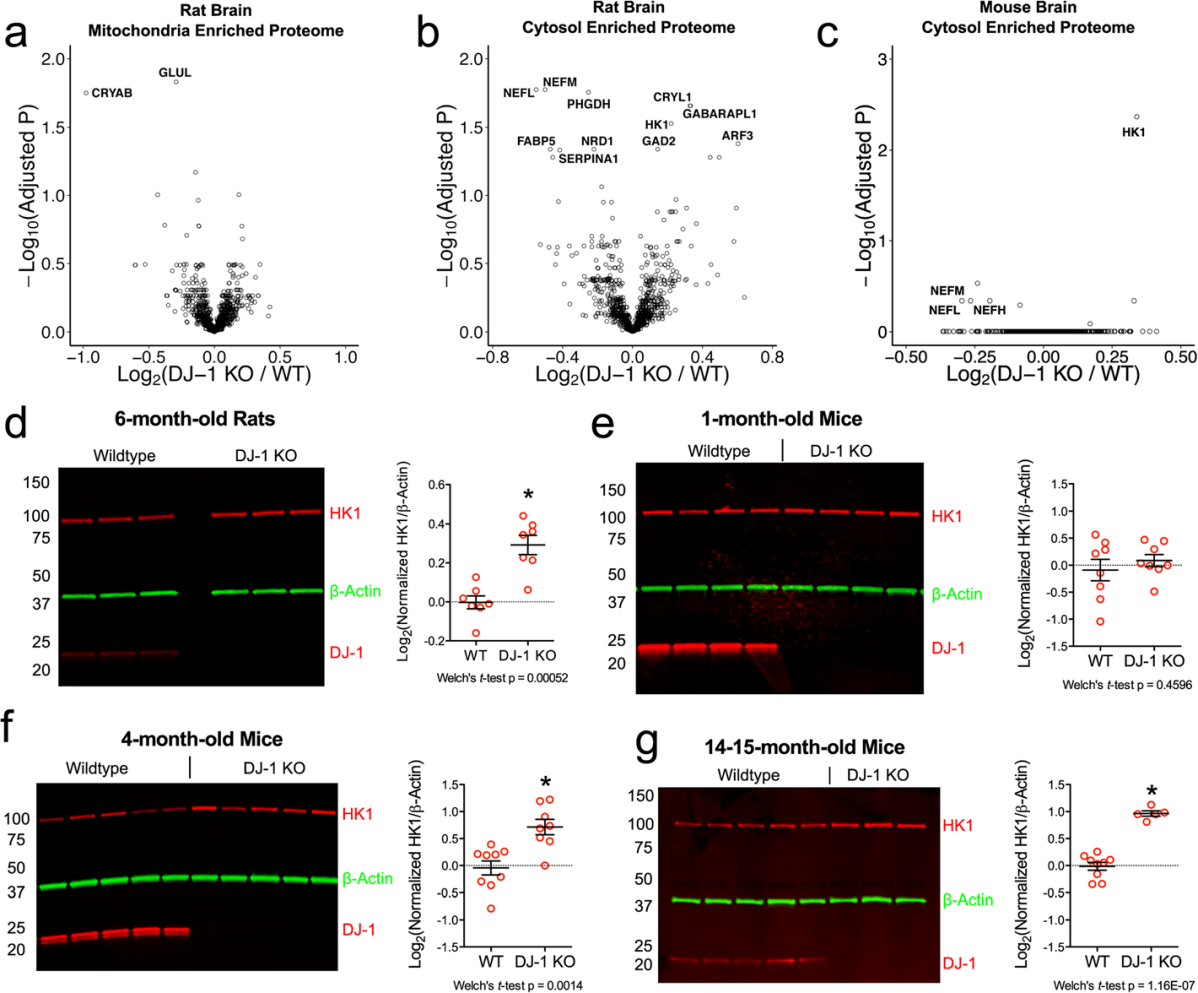
Proteomic screens identify increases in cytosolic HK1 in the brains of DJ-1 knockout rodents. **a**-**b** Volcano plots displaying abundance of 994 proteins in mitochondria-enriched fractions (**a**) and 957 proteins in cytosol-enriched fractions (**b**) of 6-month-old male rat brains (*n* = 7 WT, n = 7 DJ-1 knockout). Significantly altered proteins (Benjamini Hochberg (BH) adjusted Welch’s *t*-test *p* < 0.05) are labeled with their official gene names. The DJ-1 points were omitted from the graph for clarity but are located at (−2.237, 10.02) in (**a**) and (−2.95, 8.99) in (**b**). (**c**) Volcano plot of 742 proteins quantified in the cytosol-enriched fraction of 14–15-month-old mouse brains (*n* = 9 WT (5 M, 4 F), *n* = 5 DJ-1 knockout (3 M, 2 F)). NEFL, NEFM, and NEFH are labeled but did not reach statistical significance (BH adjusted Welch’s *t*-test p < 0.05). DJ-1 was omitted from the graph but is located at (−1.99, 3.97). (**d**-**f**) Western blots showing levels of HK1 protein in the cytosol-enriched fraction of rat brains (**d**, 6-month-old males, *n* = 7 WT, 7 DJ-1 knockout),1-month-old mouse brains (**e**, *n* = 8 WT (4 M, 4 F), *n* = 8 DJ-1 knockout (4 M, 4 F)), 4-month-old mouse brains (**f**, n = 9 WT (4 M, 5 F), *n* = 8 DJ-1 knockout (5 M, 3 F)), and 14–15-month-old mouse brains (**g**, *n* = 9 WT (5 M, 4 F), *n* = 5 DJ-1 knockout (3 M, 2 F)). β-Actin was used as a loading control. Graphs display the mean, standard error of the mean (SEM), and * indicates *p* < 0.05 (Welch’s *t*-test)

We quantified approximately 995 proteins in the mitochondrial enriched samples and found that only three were significantly changed in the DJ-1 knockout rats, including DJ-1 itself and the astrocyte-specific proteins, α-crystallin B chain (CRYAB) and glutamine synthetase (GLUL) (Fig. 1a, Additional file 1: Table S1, and ProteomeXchange: PXD005215). Additionally, 958 proteins were identified in the cytosolic fractions, of which twelve including DJ-1 were differentially expressed in WT versus DJ-1 knockout rats (Fig. 1b, Additional file 1: Table S1). Six proteins from these experiments were selected for technical validation using immunoblotting. We confirmed that there were significant differences in protein levels of glutamine synthetase, neurofilament light chain, α-crystallin B chain, glutamate decarboxylase 2 (Additional file 2: Figure S1a-e), and hexokinase-1 (Fig. 1d) in response to loss of DJ-1.

In contrast to rats, DJ-1 knockout mice do not exhibit any neurodegenerative phenotypes [31, 44, 45]. We therefore reasoned that while some of the above changes might relate to early events preceding frank neurodegeneration in rats, any differences in protein abundance between WT and knockout mice would likely be more directly related to DJ-1 deficiency than secondary events. An iTRAQ experiment performed on cytosolic brain extracts of 14–15-month-old WT and DJ-1 knockout mice allowed the quantitation of 742 proteins (Fig. 1c, Additional file 1: Table S1). From this experiment, only DJ-1 and HK1 were significantly different in protein abundance between the two genotypes. Therefore, across two rodent species the only consistent response to DJ-1 deficiency at the proteomic level that we could validate was the accumulation of HK1 in the cytosolic fraction.

To further validate these findings, the cytosolic fractions of WT and DJ-1 knockout mice across different ages were examined for HK1 levels. While no difference was observed in the 1-month-old mice (Fig. 1e), significant accumulation of HK1 was detected in DJ-1 knockout mice at 4 months of age and a numerically larger accumulation in a 14–15 month-old cohort (Fig. 1f, g). Thus, the accumulation of HK1 in the cytosol is an age-dependent phenomenon, and is not seen early in life but occurs only in older animals.

### Expression and relocalization of HK1 in the DJ-1 knockout mouse brain

**Fig. 2:**
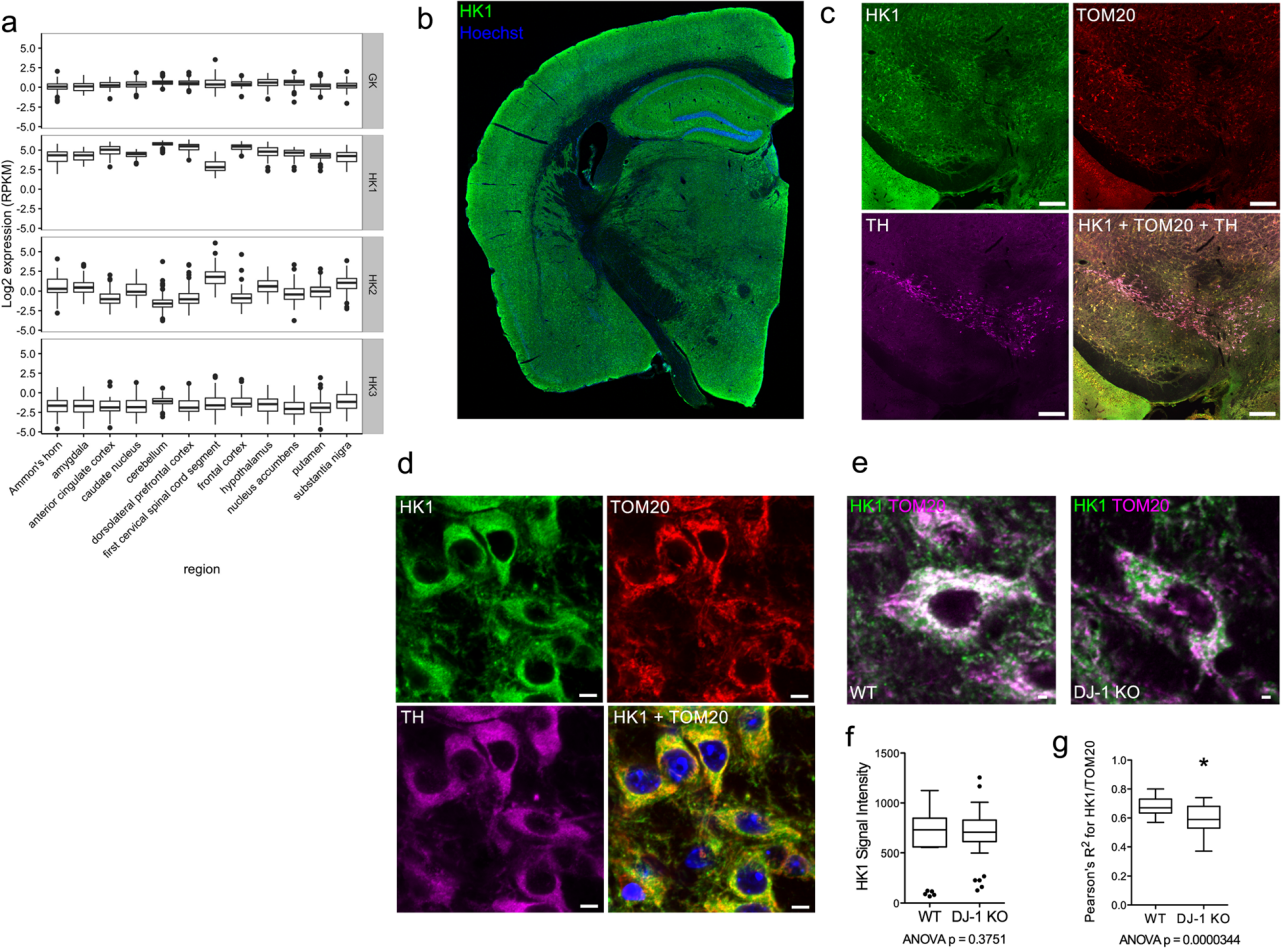
Hexokinase expression in the brain and mitochondrial detachment of HK1 caused by the loss of DJ-1. **a** Expression levels of the four hexokinase genes in different regions of the human brain based on data downloaded from GTEx. **b** HK1 immunoreactivity in a coronal section of a 1-year-old WT mouse brain shows ubiquitous expression of the protein. Scale bar: 500 μm. **c**-**d** HK1 expression in mouse dopaminergic SNpc neurons. TOM20 stains for mitochondria, and tyrosine hydroxylase (TH) is a marker for dopaminergic neurons. Scale bar **c**: 200 μm, Scale bar **d**: 5 μm. **e** High-magnification images of individual TH-positive neurons in the SNpc of a 1-year-old WT mouse and a 1-year-old DJ-1 knockout mouse. HK1 is shown in green, the mitochondrial marker TOM20 in pink, and white pixels indicate overlap of the two signals. Scale bar: 2 μm. **f** HK1 signal intensity in dopaminergic SNpc neurons of 1-year-old mice (*n* = 5 WT (1 M, 4 F), *n* = 7 (3 M, 4 F) DJ-1 knockout). The intensity values of five neurons from each mouse (*n* = 25 WT cells, *n* = 35 DJ-1 knockout cells) are displayed in the boxplot with whiskers drawn according to the Tukey method and outliers shown as black dots. The data were fitted to a linear model and the *p* value of an ANOVA testing for the effect of genotype is displayed below the graph. **g** HK1 and TOM20 signal colocalization in dopaminergic SNpc neurons of 1-year-old mice. The data come from the same cells as (**f**), and are graphed and compared as (**f**) with * indicating *p* < 0.05

DJ-1 is widely expressed across different brain regions and is found in most cell types of the CNS, including neurons and astrocytes [46]. The hexokinase family of proteins consists of HK1, hexokinase 2 (HK2), hexokinase 3 (HK3), and glucokinase (GK), whose expression varies among tissues [47]. Using publically available data from the GTEx consortium [48], we found that HK1 is highly expressed across most regions of the human brain, whereas the other three isoforms are expressed at low or undetectable levels (Fig. 2a). This is consistent with our prior data comparing mouse brain and human cancer cell lines [41], which also suggested that HK1 is the major hexokinase in the brain.

A second cohort of 1-year-old WT and DJ-1 knockout mice [39] distinct from the one used for the iTRAQ proteomics experiment was used for a series of immunohistochemical experiments. We validated a mouse HK1 monoclonal antibody by verifying that it detected a single band on immunoblots (Additional file 3: Figure S2a) and showed a similar pattern of staining in primary cultures as other commercial anti-HK1 antibodies (Additional file 3: Figure S2b, c). All major cell types in various regions of the mouse brain were positively stained for HK1 (Fig. 2b). Strong HK1 immunostaining was present in the dopaminergic neurons of the substantia nigra pars compacta (SNpc) (Fig. 2c, d) and the Orexin-A-positive, glucose-sensing neurons in the lateral hypothalamus (Additional file 4: Figure S3a). In contrast, HK1 expression was below detection levels in cerebellar Purkinje cells (Additional file 4: Figure S3b), in agreement with earlier reports [49]. Astrocytes expressing HK1 could easily be identified in white matter tracts (Additional file 4: Figure S3c, d), and IBA1-positive microglia also expressed HK1 (Additional file 4: Figure S3e). Therefore, the major brain hexokinase is HK1 and this protein is expressed in most, but not all, principal cell types in many brain regions.

Because of their potential relevance to PD, we used SNpc dopaminergic neurons to determine whether there were differences in HK1 localization consequential to DJ-1 deficiency. A partial colocalization of HK1 with the mitochondrial marker TOM20 was observed in TH-positive neurons in WT animals (Fig. 2d). No difference in total HK1 staining was found in SNpc neurons from WT and DJ-1 knockout mice (Fig. 2e, f); however, the degree of colocalization between HK1 and TOM20 was significantly lower in DJ-1 knockout mice compared to WT animals (Fig. 2g). Therefore, it appears that the accumulation of HK1 in cytosolic brain fractions in response to DJ-1 deficiency (Fig. 1) results from HK1 dissociation from mitochondria.

### Accumulation of polyols in DJ-1 knockout mouse brain

The association of hexokinases with mitochondria is an important signaling event that integrates cellular survival with metabolic events [50]. We therefore asked if additional high-content unbiased approaches could be applied to test whether DJ-1 deficient mice have distinct metabolic profiles as compared to WT animals.

**Fig. 3:**
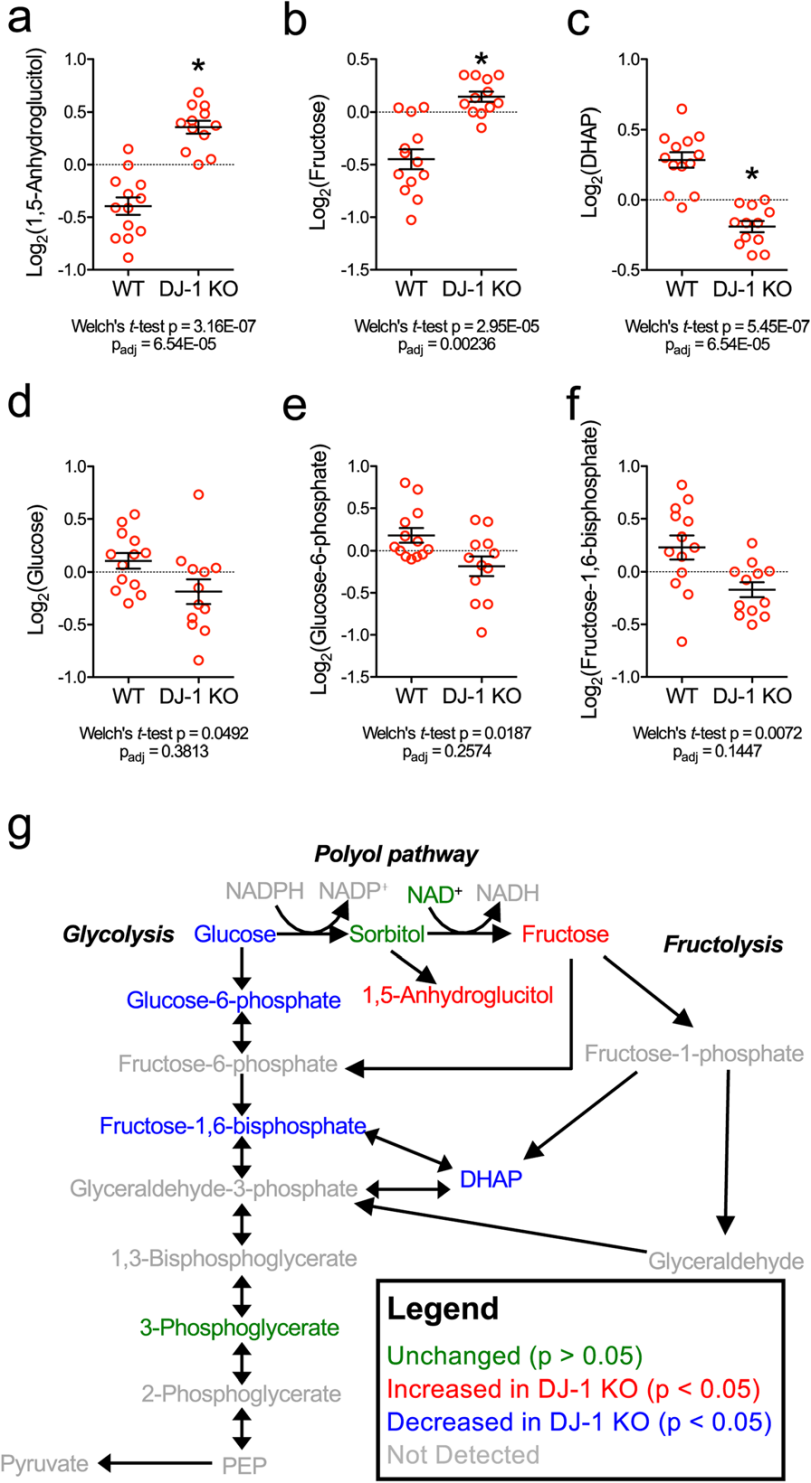
Activation of the polyol pathway of glucose metabolism in the DJ-1 knockout mouse brain. **a**-**f** Graphs showing levels of 1,5-anhydroglucitol, fructose, DHAP, glucose, glucose-6-phosphate, and fructose-1,6-bisphosphate in WT and DJ-1 knockout mouse brains. Metabolite levels were determined using a high-content metabolomics screen of 240 biochemicals in 4-month-old mouse brains (*n* = 13 WT (8 M, 5F), *n* = 12 DJ-1 knockout (6 M, 6F)). The results of a Welch’s *t*-test with and without correction for multiple tests using the BH method are displayed beneath each graph. * indicates p_adj_ < 0.05. **g** Summary of alterations in glucose metabolites found in the DJ-1 knockout mouse brain. Note that colors indicate significance before correction for multiple testing

The abundance of 240 biochemicals extracted from the brains of 4-month-old DJ-1 knockout and WT mice was measured (Additional file 5: Table S2 and Metabolomics Workbench: ST000260). Significant differences in the concentrations of five metabolites were found between the two genotypes after correction for multiple testing. In addition to the polyols 1,5-anhydroglucitol and fructose, which are two metabolites of sorbitol (Fig. 3a, b), there was a significant decrease in dihydroxyacetone phosphate, a glycolytic intermediate (Fig. 3c). A significant reduction in the levels of glucose, glucose 6-phosphate, and fructose 1,6-bisphosphate in the DJ-1 knockout brains was observed but only without multiple testing correction (unadjusted *p* < 0.05; Fig. 3d-f). These observations led us to question whether changes in glucose levels could be observed outside of the brain in DJ-1 knockout rodents, but we did not observe any differences in blood glucose levels in either fed or fasted 14–15 month old mice (Additional file 6: Figure S4). Collectively, these results are consistent with the hypothesis that the loss of DJ-1 is accompanied by an impaired brain metabolite profile where glucose is shunted away from glycolysis and into the polyol pathway (Fig. 3g). These data are further consistent with lower HK1 activity due to partial dissociation from mitochondria [47, 51] as shown in our proteomics data.

### Dysfunctional AKT signaling in the DJ-1 knockout rat brain

**Fig. 4:**
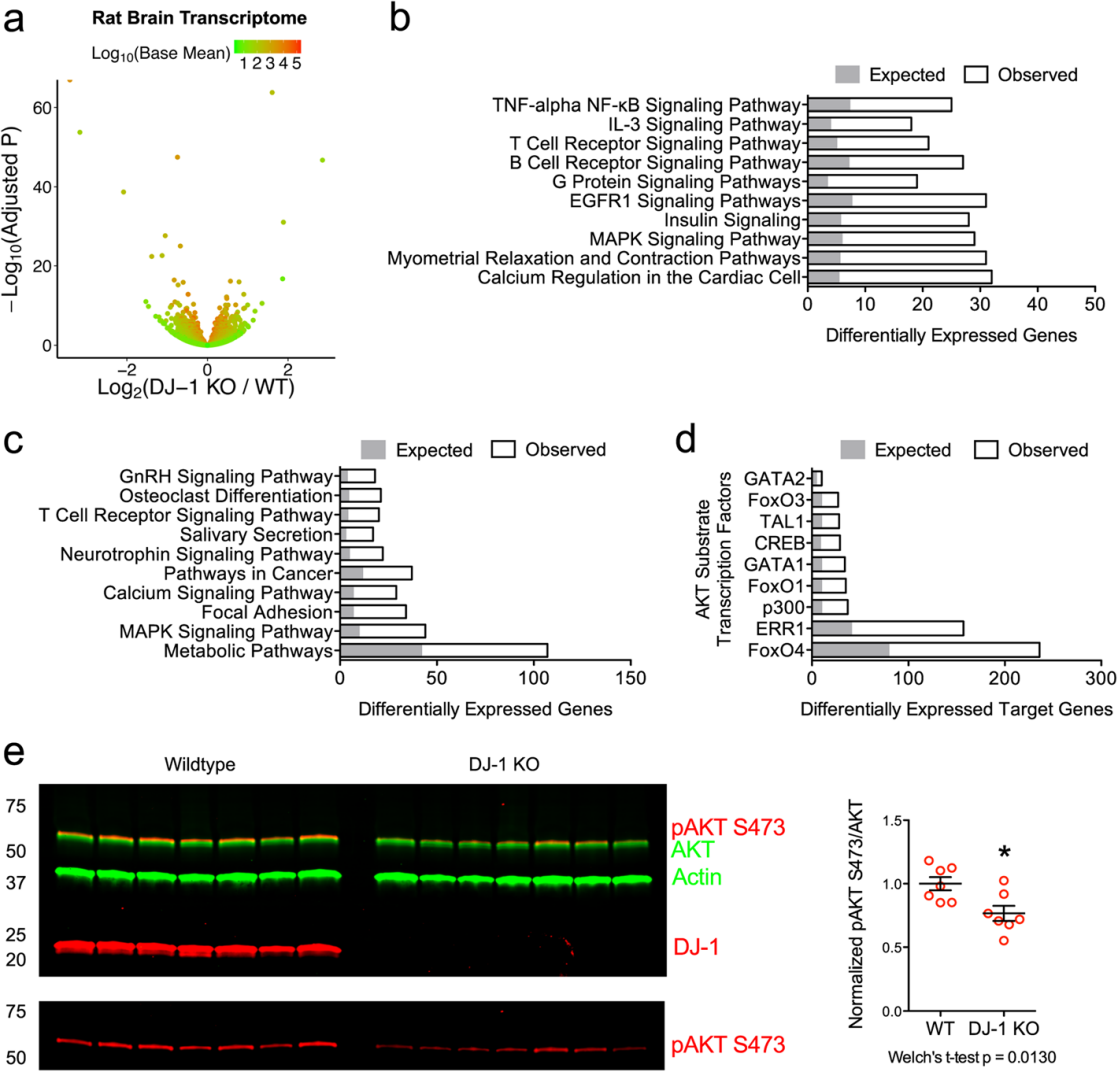
RNA-Seq profile of the DJ-1 knockout rat brain demonstrates altered AKT signaling. **a** Effect of DJ-1 depletion as illustrated by volcano plot of 15,537 mRNA transcripts quantified using RNA-Seq in mRNA isolated from 6-month-old male rat brains (*n* = 8 WT, *n* = 6 DJ-1 knockout). Over 2000 genes are differentially expressed in the DJ-1 knockout rat brains (BH adjusted p < 0.05). Genes are colored by the log_10_ of the base mean expression. **b**-**c** Top 10 Wikipathways (**b**) and KEGG pathways (**c**) from the significant enrichment of differentially expressed mRNAs in the DJ-1 knockout:WT RNA-Seq comparison. The number of genes expected to be in the list by random chance is indicated in grey and the entire bar indicates the observed numbers. **d** AKT-regulated downstream transcription factors that activate target genes, leading to significant overrepresentation of differentially expressed mRNAs present in DJ-1 knockout compared to WT rat brains. Expected and observed number of genes are indicated as in (**b**) and (**c**). **e** Western blot showing the levels of total AKT, pAKT S473, DJ-1, and β-actin in whole-brain homogenates from 6-month-old male rats (*n* = 7 WT, *n* = 7 DJ-1 knockout). The ratio of pAKT S473 to total AKT was normalized and graphed as in Fig. 1d. *, *p* < 0.05

One potential limitation of the proteomics data generated above is that it had relatively modest coverage, with approximately 1000 proteins measured compared to the estimated ~28,000 genes expressed in the brain [52]. In order to elucidate the mechanisms implicated in the relocalization of HK1 and improve coverage of events in the brains of DJ-1 deficient rats, we performed RNA-Sequencing (RNA-Seq) to quantify mRNA from the remaining hemisphere of DJ-1 knockout brains that were used in the proteomic experiments. Rats were chosen because of the greater number of events in the proteomics experiments. Of the 15,537 transcripts identified for quantification, 2072 genes were differentially expressed (Fig. 4a, Additional file 7: Table S3, and GEO: GSE71968), with many of these representing members of several related pathways that depend on signaling through the serine/threonine kinase AKT (Fig. 4b, c).

A search for differentially expressed genes encoding known targets of various transcription factors revealed the enrichment of 9 different AKT substrates, including three members of the Forkhead box O (FoxO) family (Fig. 4d). Both the gene set enrichment and transcription factor binding target analyses suggested that the loss of DJ-1 had a negative impact on AKT signaling, a nexus for signaling and gene expression changes in the rodent brain. Consistent with this hypothesis, immunoblot of whole brain lysates with an antibody recognizing the active form of AKT (pSer-473) showed significantly lower phospho-active AKT in the brains of DJ-1 knockout compared to WT rats (Fig. 4e).

**Fig. 5:**
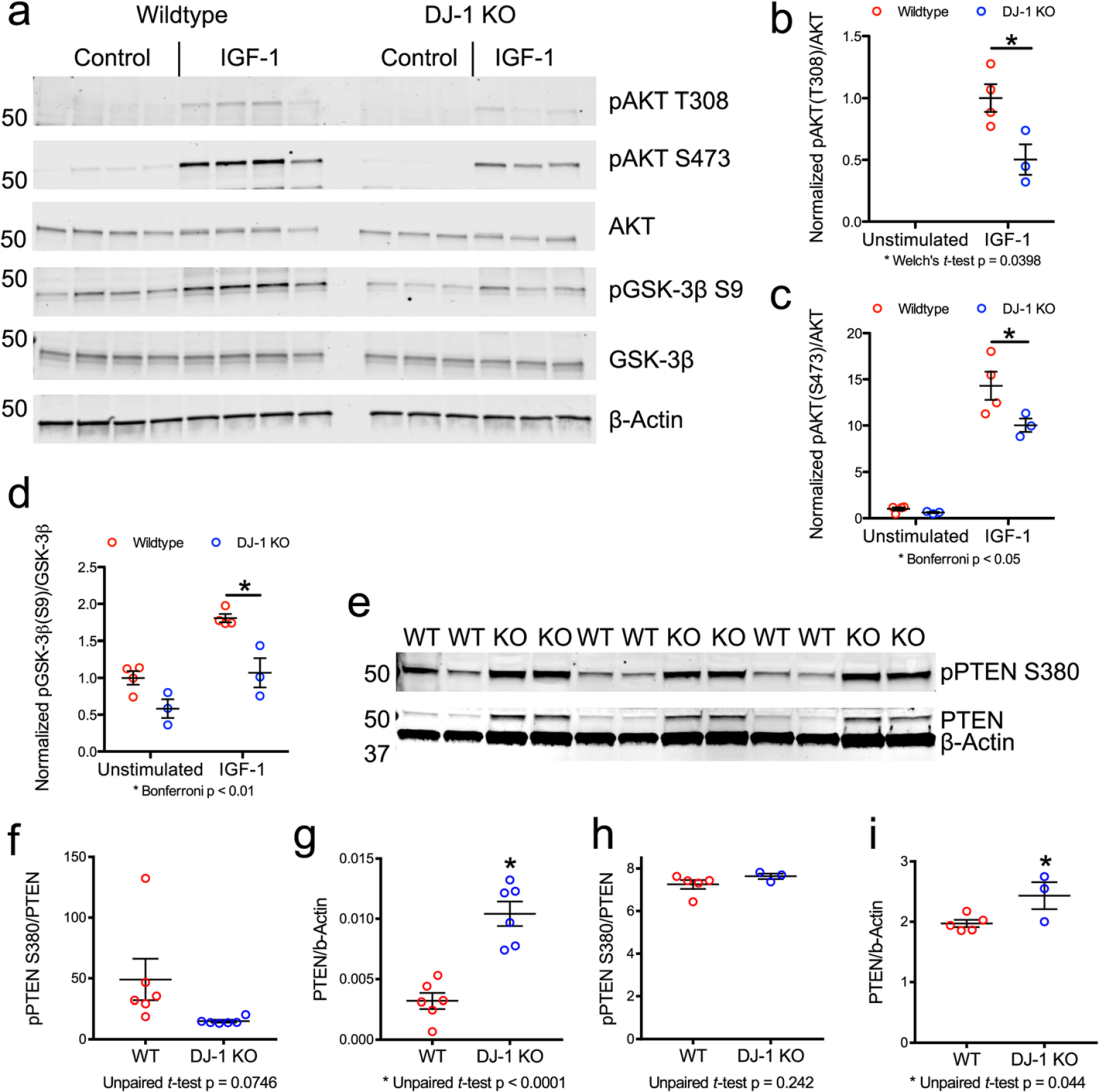
Effect of DJ-1 deficiency on the PTEN/PI3K/AKT/GSK3β signaling cascade. **a** Western blots showing the levels of pAKT T308, pAKT S473, and pGSK-3β S9 in primary astrocytes from WT and DJ-1 knockout mice following a 5- min stimulation with IGF-1 (*n* = 4 WT, *n* = 3 DJ-1 knockout, cultures taken from separate animals). Total levels of AKT, GSK-3β, and β-actin are also shown. **b**-**d** Quantification of pAKT T308 (**b**), pAKT S473 (**c**), and pGSK-3β S9 (**d**) from the blot shown in (**a**). pAKT T308 was not detected in unstimulated cells and a Welch’s *t*-test was used to assess the IGF-1 response (**b**). Two-way ANOVA followed by Bonferroni’s multiple comparison test was used in (**c**) and (**d**). **e**-**g** Western blots and quantification for PTEN and pPTEN S380 in primary mouse astrocytes from WT and DJ-1 knockout mice (*n* = 6 WT, *n* = 6 DJ-1 knockout, experiment performed using technical replicates). **h**-**i** PTEN and pPTEN S380 levels in total brain lysates from 14 to 15-month-old WT and DJ-1 knockout mice determined by immunoblot (*n* = 5 WT, *n* = 3 DJ-1 knockout). Unpaired t-tests were used to compare groups shown in (**e**-**i**)

To test whether DJ-1 might functionally interact with AKT, we treated primary astrocytes with the growth factor IGF-1. As expected, this increased the levels of phospho-active AKT and the inactivating phosphorylation of glycogen synthase kinase 3β (GSK-3β) on serine-9, a major downstream target of AKT [53]. In contrast to wild type cells, DJ-1 deficient astrocytes were refractory to IGF-1 stimulation (Fig. 5a-d). Since DJ-1 has been shown to negatively regulate PTEN [11–13], the major antagonist of PI3K/AKT signaling, we assayed total and phosphorylated PTEN levels in the astrocytes. We found no differences in phosphorylation of S380 (a marker of PTEN activity [54]), but did observe increased total PTEN levels in the DJ-1 knockout cells (Fig. 5e-g). Similar results were observed in a cohort of aged mice (Fig. 5h, i).These results support the notion that AKT is a downstream target of DJ-1 both in vivo and in vitro, likely due to DJ-1 dependent regulation of PTEN.

**Fig. 6:**
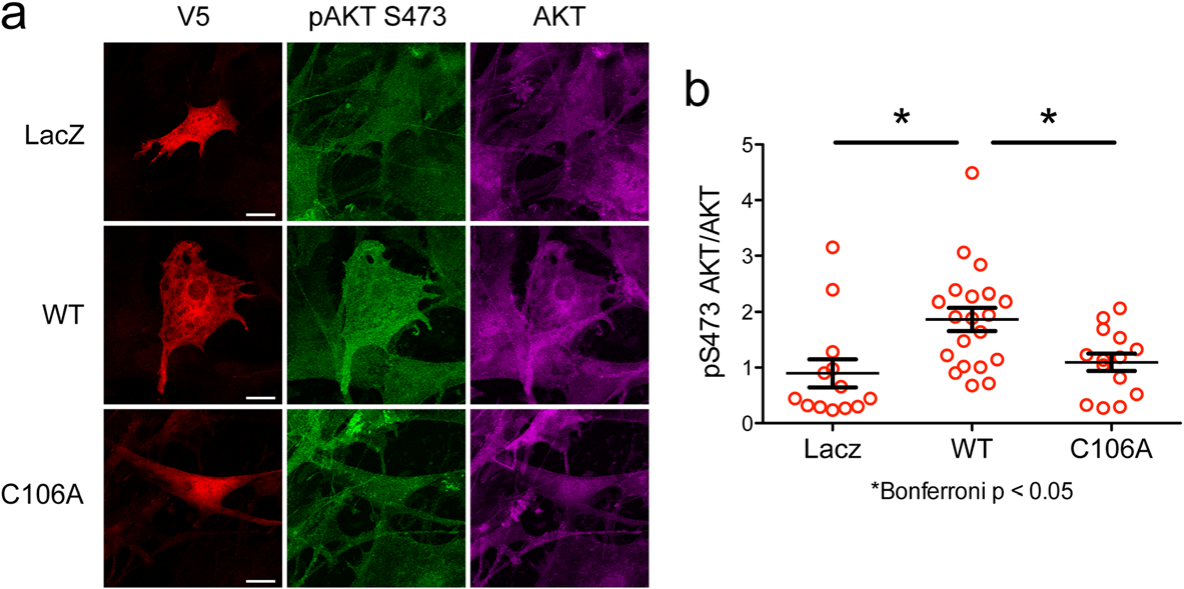
DJ-1 C106 is necessary for regulation of AKT. Primary DJ-1 knockout mouse astrocytes were transfected with LacZ-V5, wildtype DJ-1-V5, and C106A DJ-1-V5 and stimulated with IGF-1 for 5 min. **a** Immunocytochemistry for V5 (red), pAKT S473 (green), and total AKT (pink). Scale bar: 20 μm. **b** Quantification of pAKT S473 normalized to total AKT (*n* = 13 LacZ cells, *n* = 20 wildtype DJ-1 cells, *n* = 14 C106A DJ-1 cells). Groups were compared using One-way ANOVA followed by Bonferroni’s multiple comparison test

Cysteine 106 in DJ-1 is sensitive to oxidation and critical for the neuroprotective effects of DJ-1 [7]. We assessed whether or not C106 was necessary for DJ-1 regulation of AKT signaling by transfecting wildtype DJ-1 and C106A DJ-1 into DJ-1 knockout astrocytes and then stimulating them with IGF-1. Using immunocytochemistry to probe AKT phosphorylation in cells expressing the exogenous proteins, we found that wildtype DJ-1 increased pAKT compared to both LacZ control and C106A transfected cells (Fig. 6a, b). This experiment demonstrates, first, expression of human DJ-1 can rescue the effects of DJ-1 knockout in mouse cells and, second, that C106 is critical for the ability of DJ-1 to modify AKT signaling.

### Cytosolic accumulation of hexokinase inhibits the PINK1/parkin pathway

**Fig. 7:**
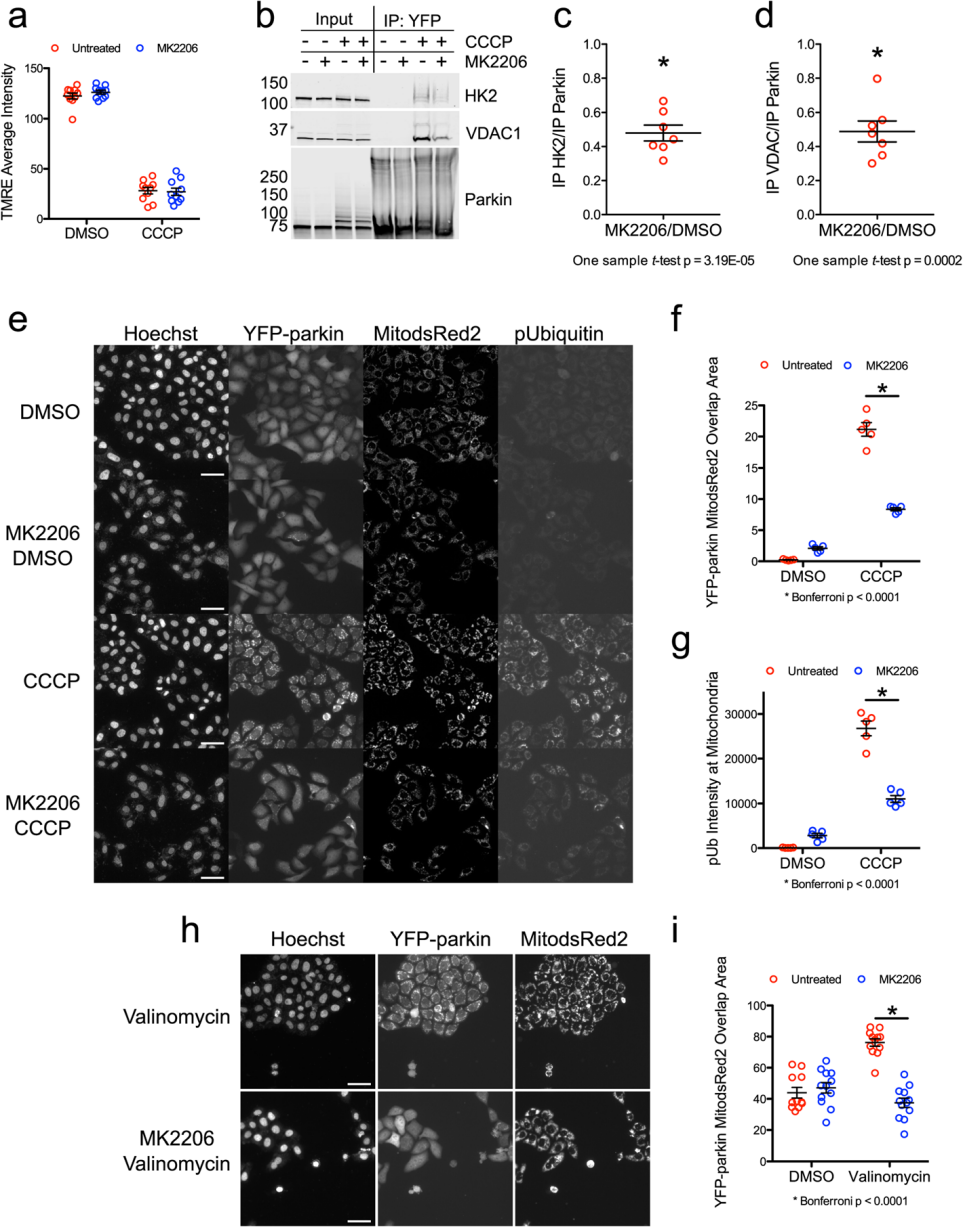
AKT signaling promotes PINK1/parkin pathway activation. **a** Mitochondrial membrane potential after overnight incubation with MK2206 in HeLa cells with and without the protonophore CCCP. **b**-**d** AKT was inhibited in YFP-parkin expressing HeLa cells using MK2206 (10 μM) for 24 h prior to a 1-h treatment with 10 μM CCCP and then YFP-parkin was immunoprecipitated. Levels of immunoprecipitated HK2, VDAC1, and parkin proteins are shown in (**b**) and quantified in (**c**) and (**d**) (n = 7 replicates done over the course of 4 separate experiments, one sample *t*-test comparing MK2206/DMSO versus 1). **e**-**g** HeLa cells stably expressing YFP-parkin and MitodsRed2 were treated with MK2206 (10 μM) prior to 1-h treatment with CCCP (5 μM) and cells were analyzed using high-content imaging. Photomicrographs of Hoechst (nuclei), YFP-parkin, MitodsRed2, and pUbiquitin S65 are shown in (**e**). Quantification of the overlap between YFP-parkin and MitodsRed2 is shown in (**f**), while the intensity of pUbiquitin S65 in mitochondria is displayed in (**g**) (*n* = 400 cells/well, 5 wells per condition). Two-way ANOVA with Bonferroni’s multiple comparison test was used to compare groups. Scale bar: 50 μm. **h** HeLa cells stably expressing YFP-parkin and MitodsRed2 were treated with MK2206 prior to 2-h treatment with valinomycin (10 μM). **i** Quantification of YFP-parkin overlap with MitodsRed2 from (**h**) (*n* = 400 cells/well, 11–12 wells per condition). Comparison and scale bar as shown in (**e**-**g**)

We have shown that DJ-1 accumulates in the cytosolic fractions of rodent brains after aging, and this is associated with accumulation of PTEN, a regulator of signaling through the PI3K/AKT pathway. Earlier findings from our laboratory, also generated using unbiased screens, indicated the requirement of hexokinase activity in the recruitment of parkin to depolarized mitochondria in HeLa cells [41]. To extend these observations, we treated HeLa cells with the AKT inhibitor MK2206, which should mimic loss of DJ-1 seen in aging animals. In contrast to the brain, HeLa cells primarily express HK2 [41], and we found that MK2206 partially prevented the formation of a parkin/VDAC/HK2 protein complex in response to mitochondrial depolarization without affecting the degree of depolarization caused by the protonophore CCCP (Fig. 7a-d). MK2206 also significantly blocked the accumulation of parkin on mitochondria (Fig. 7e, f) and prevented ubiquitin phosphorylation in response to mitochondrial depolarization by CCCP (Fig. 7e, g), consistent with an AKT-mediated activation of both PINK1 and parkin. We also treated cells with MK2206 then valinomycin, a potassium ionophore that triggers parkin translocation to mitochondria and subsequent apoptosis [55]. The combination of MK2206 and valinomycin resulted in the appearance of apoptotic nuclei and blebbing cells, indicating that AKT inhibition sensitized the cells to valinomycin induced apoptosis (Fig. 7h, Hoechst and YFP-parkin panels). Importantly, MK2206 also blocked the translocation of parkin to mitochondria in the surviving cells (Fig. 7h, i).

**Fig. 8:**
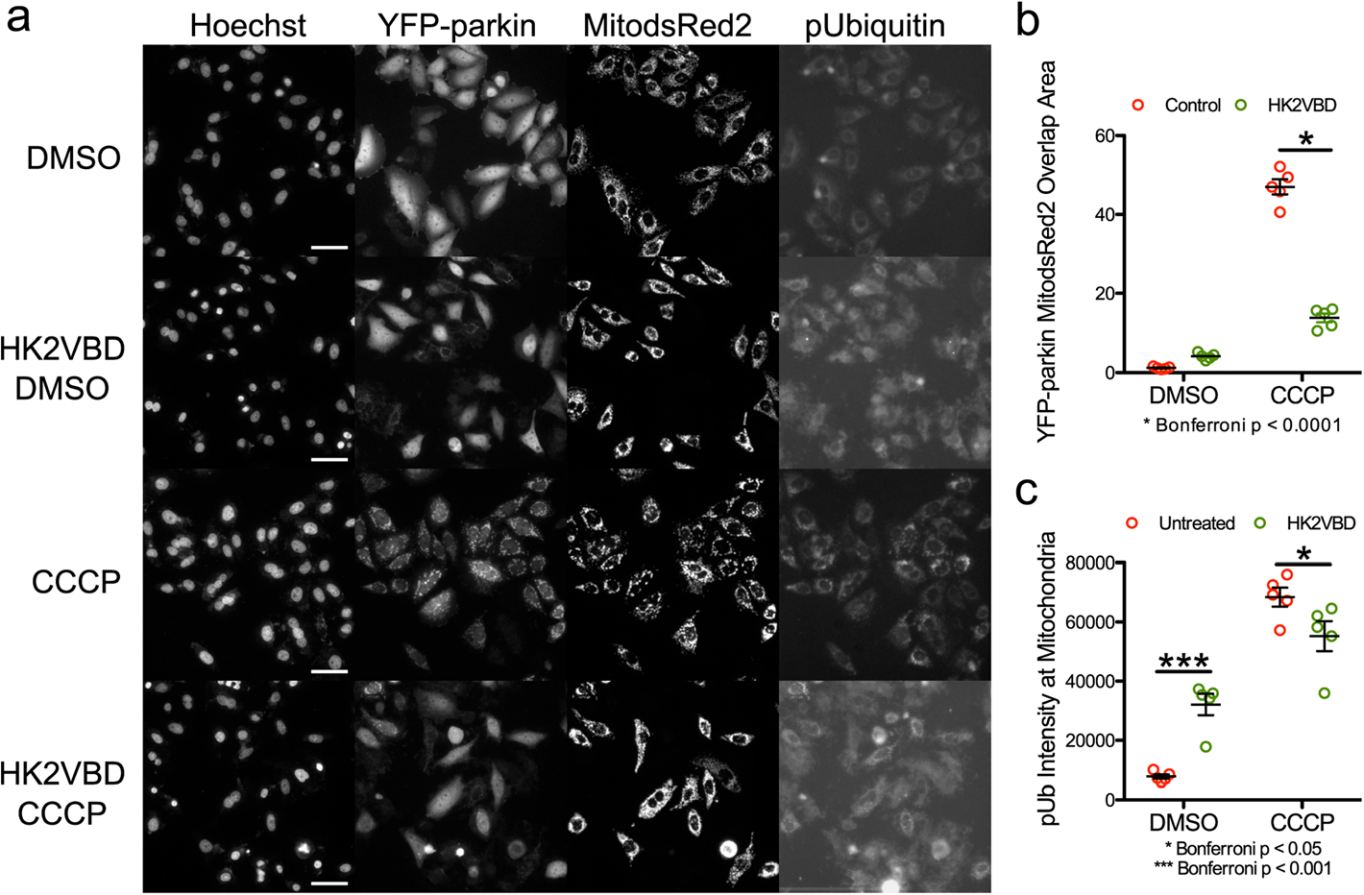
Cytosolic accumulation of HK2 inhibits the PINK1/parkin pathway. **a**-**c** HK2 was dissociated from mitochondria in HeLa cells using a peptide derived from the VDAC-binding domain of HK2 (HK2VBD) for 30 min prior to a 1-h treatment with 5 μM CCCP. Images and quantification are displayed as in Fig. 6

To test whether accumulation of cytosolic hexokinases would block PINK1/parkin activation, we used a cell-permeable peptide containing the VDAC-binding domain of HK2 (HK2VBD) to dissociate HK2 from mitochondrial VDAC. Incubation of HeLa cells with the peptide resulted in some cell death with and without MK2206 (Fig. 8a, Hoechst panels), which is consistent with the pro-apoptotic effects of dissociating HK2 from VDAC [56]. Moreover, the increased translocation of parkin and ubiquitin phosphorylation in response to CCCP were significantly reduced by a 30-min pretreatment with the HK2VBD peptide (Fig. 8a-c). Interestingly, the HK2VBD peptide increased the phosphorylated ubiquitin levels in cells treated with DMSO, which is expected given the reports that the peptide depolarizes mitochondria [56]. Taken together, these results show that either inhibiting AKT or dissociating HK2 from VDAC prevents a robust activation of the PINK1/parkin pathway in cells with depolarized mitochondria.

## Discussion

**Fig. 9:**
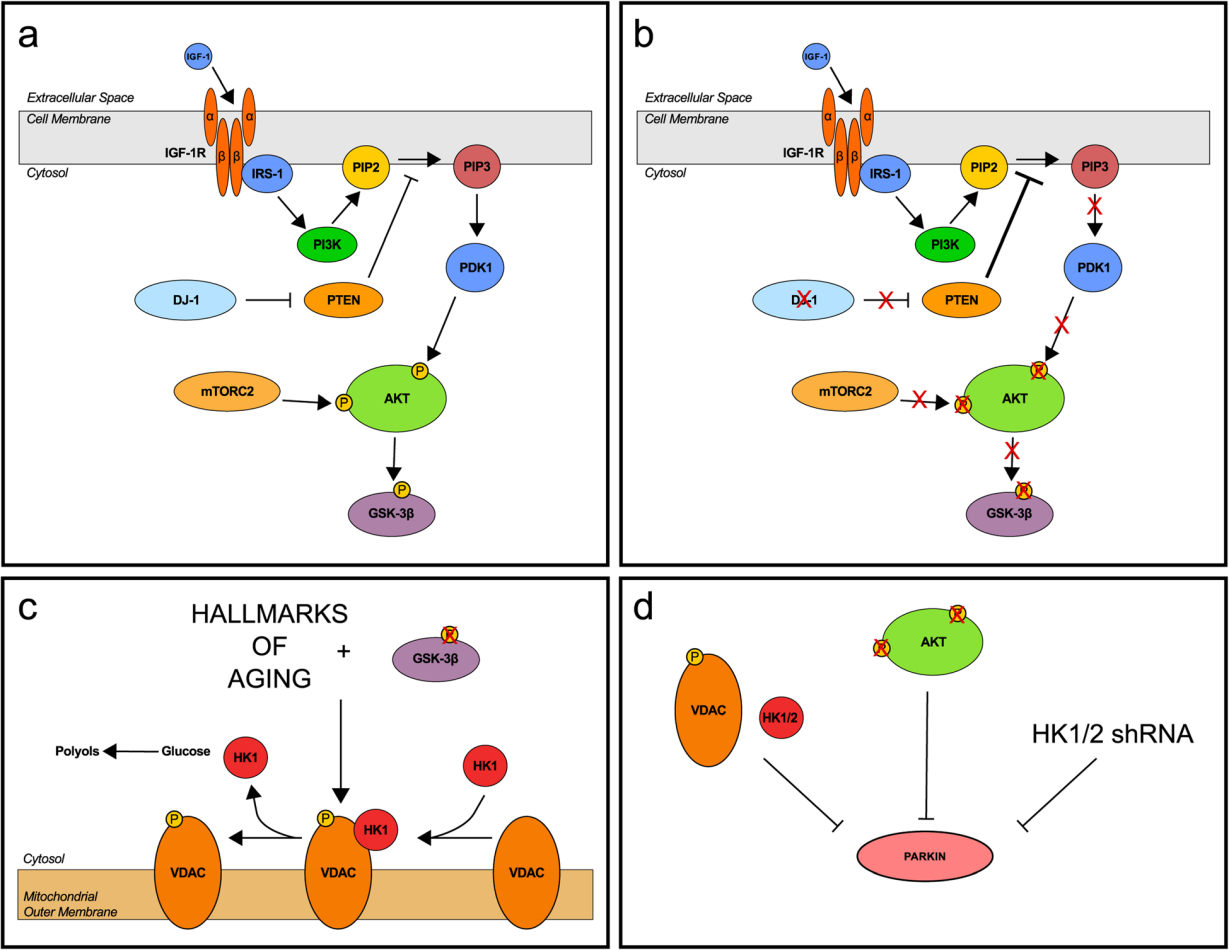
The hexokinases link DJ-1 to the PINK1/parkin pathway. **a** Normal signaling through the PI3K/AKT pathway. DJ-1 negatively regulates PTEN which promotes signal transduction to AKT. **b** PI3K/AKT signaling in the absence of DJ-1. Without negative regulation by DJ-1, PTEN inhibits signaling to AKT. **c** The effect of the loss of DJ-1 on PI3K/AKT/GSK-3β combined with the hallmarks of aging (e.g. deregulated nutrient sensing and mitochondrial dysfunction) leads to the buildup of HK1 in the cytosol and activation of the polyol pathway in the brains of aged rodents. **d** Promoting the dissociation of hexokinases from VDAC, inhibiting AKT, and decreasing hexokinase mRNA all inhibit parkin translocation to depolarized mitochondria in immortalized cells. For details regarding HK1/2 shRNA and other AKT inhibitors aside from MK2206, please see [41]

Using a series of unbiased high-content approaches to measure thousands of biomolecules in the brains of two DJ-1 deficient rodent models, we have found that the single primary effect caused by the loss of DJ-1 is an age-dependent redistribution of HK1 from mitochondria to the cytosol. This is accompanied by a shift in glucose metabolism from glycolysis to the polyol pathway, and can be explained by the effect of DJ-1 on the PTEN/AKT/GSK-3β signaling cascade (Fig. 9a-c). Importantly, the negative outcomes of DJ-1 deficiency on AKT and hexokinase trafficking lead to significant inhibition of the PINK1/parkin pathway in cellular models (Fig. 9d) [41]. Our findings demonstrate the cardinal function of hexokinases in linking DJ-1 to the PINK1/parkin pathway, and suggest a complex, age-dependent impact of DJ-1 loss to PINK1/parkin pathway inhibition in vivo.

Multiple functions have been ascribed to DJ-1 and it has been difficult to clarify which, if any, have physiological relevance. Based on the current results, we conclude that the ability of DJ-1 to inhibit PTEN and, therefore, promote AKT signaling is the dominant response in vivo. It has been found previously that DJ-1 is a negative regulator of PTEN using a genetic screen in Drosophila [12], and a mediator of AKT signaling in vivo [13, 57, 58]. One proposed direct mechanism by which DJ-1 may inhibit PTEN is via transnitrosylation [11], although more work is required to independently validate this observation. Another mechanism that has been observed in brown adipose tissue is the DJ-1 dependent regulation of PTEN protein degradation [13], and our data suggest that this may also occur in the brain.

AKT signaling promotes the attachment of HK1 and HK2 to the mitochondrial outer membrane via the voltage-dependent anion channel (VDAC) [59]. AKT can directly phosphorylate HK2 on threonine-473 which increases binding to VDAC [60]. However, because HK1 does not contain a complete AKT consensus sequence, a more plausible mechanism is that AKT activation inhibits GSK-3β by phosphorylation at serine-9 [53], which, in turn, would prevent GSK-3β from phosphorylating VDAC and hence disrupt interaction with HK1 [61]. Our proteomics data would support a disrupted HK1 association with VDAC driven by changes in AKT signaling.

By binding to VDAC, HK1 has preferential access to a pool of intramitochondrial ATP to phosphorylate glucose [47]. However, in the absence of DJ-1, excessive AKT inhibition by PTEN results in a buildup of HK1 in the cytosol where it must compete for extramitochondrial ATP. Increased formation of polyols ensues, consistent with our metabolomics data. This concept is supported by the finding of a positive correlation between levels of cytosolic HK1 and polyols in human brains with psychiatric illnesses [51].

DJ-1 is responsive to oxidative stress and protects cells against mitochondrial stress [7, 62]. Cultured cells lacking DJ-1 have repeatedly been shown to have increased oxidative stress and fragmented mitochondria [29, 63–65]. Furthermore, deletion of DJ-1 increases mitochondrial oxidative stress in SNpc neurons in living mice [66]. The detachment of hexokinases from mitochondria results in increased oxidative stress, decreased mitochondrial membrane potential, and sensitizes cells to apoptosis [56, 67], observations that we have confirmed here in cells with ectopic expression of parkin. Therefore, it is possible that the effects of DJ-1 on mitochondria are partly due to disrupted interactions between hexokinases and mitochondria.

However, we have yet to observe strong DJ-1 dependent effects on hexokinases in cell culture models despite them having measurable defects in PTEN/PI3K/AKT signaling. There may be several reasons for the differences between brain and cells. The Warburg effect, which drives aerobic glycolysis in HeLa and other cancer cells, is thought to be driven by increases in HK2 and altered AKT signaling [50]. We hypothesize that any effects of DJ-1 on AKT and hexokinases in HeLa cells would be overshadowed by the Warburg effect, and that this likely explains why we and others have not observed any effects of the loss of DJ-1 on PINK1/parkin using HeLa cells [41, 68]. Additionally, aging plays a significant role in the effects of DJ-1 on HK1 distribution in the brain (Fig. 1e-g).Two of the hallmarks of aging are deregulated nutrient sensing and mitochondrial dysfunction [30]. As the brain ages, IGF-1 signaling becomes progressively dysfunctional [69]. Glucose hypometabolism is also associated with brain aging and age-related diseases [70], and it was recently reported that aerobic glycolysis decreases with normal brain aging in humans [71]. Therefore, we hypothesize that the effects of DJ-1 on hexokinase distribution are only unmasked as the brain ages. Primary cells lack these important age-related factors, and therefore are not an ideal system to investigate the interaction between DJ-1, PINK1, and parkin with regards to hexokinases. This could help reconcile the observation that the loss of DJ-1 promotes the PINK1/parkin pathway in primary neurons in a manner linked to oxidative stress [72].

Some inferences can be made about the relationship between DJ-1, PINK1, and parkin from other in vivo work. Dopaminergic neurons in the parkin knockout mouse SNpc degenerate, whereas those in DJ-1 deficient mice do not, when the mice are crossed with Polg mutator mice [39, 73]. This suggests that DJ-1 is not required for parkin mediated protection against mitochondrial DNA damage in the mouse brain*.* Additionally, experiments in Drosophila have shown that DJ-1 mutations can partially phenocopy PINK1 and parkin mutations but only with aging [28]*.* It seems likely that DJ-1 and PINK1/parkin mainly act in parallel [28, 29], but that the hexokinases represent one intersection between the two pathways. Future experiments will be required to test if, in the mammalian brain, altered PTEN/AKT signaling is required for the effects of DJ-1 deficiency on hexokinase regulation and to tie these events to the activity of the PINK1/parkin pathway. It will also be important to determine what age-dependent signals are necessary to provoke dysregulation of hexokinases.

Additionally, it is important to note that there are other observations in the literature that might indirectly support the idea that different PD genes may be related in a manner that depends, in part, on HK signaling. For example, parkin regulates the activity of Bax [74, 75], a pro-apoptotic Bcl-2 family member. An important non-metabolic effect of hexokinase dissociation from mitochondria is to promote apoptosis [56, 76, 77], likely because HK and Bax compete for binding to VDAC [78]. Thus, DJ-1 deficiency would prime cells in a pro-cell death state due to an age-dependent hexokinase accumulation in the cytosol, whereas parkin-mediated Bax turnover might partially mitigate this effect. Furthermore, hexokinases are direct substrates of parkin after triggering mitophagy with strong depolarizing signals [79, 80]. Therefore, it is expected that DJ-1 and parkin would have complex inter-relationships influenced by hexokinase localization, with parkin capable of both mitigating effects of hexokinase by ubiquitylating Bax or potentially exacerbating them by turnover of mitochondrial hexokinases.

## Conclusions

Overall, these results demonstrate how the application of mutliple integrated high-content approaches can be helpful in dissecting complex pathways related to gene mutations in vivo. In the specific context of highly conserved proteins, the comparison of different species was particularly helpful in isolating relatively primary effects from secondary changes. The use of such approaches provides a testable mechanistic hypothesis as to how phenotypes associated with DJ-1 mutations show age-dependent penetrance and partially resolve the complex relationships between different PD-associated pathways.

## Additional files

Additional file 1: Table S1. Complete list of proteins quantified using iTRAQ in DJ-1 knockout rodent brains (XLSX 309 kb)

Additional file 2: Figure S1. Technical validation of iTRAQ hits in the DJ-1 knockout rat brain by immunoblotting. (**a**-**b**) Western blots for GLUL and CRYAB in mitochondria-enriched fractions from 6-month-old DJ-1 knockout rat brains. The mitochondrial protein HSP60 was used as a loading control. Data are graphed as in Fig. 1d. (**c**-**e**) Western blots for NEFL, GAD2, and PHGDH in cytosol-enriched fractions from 6-month-old DJ-1 knockout rat brains with β-actin as a loading control. Data are graphed as in Fig. 1d. (TIFF 514 kb)

Additional file 3: Figure S2. Validation of a monoclonal anti-HK1 antibody for use in immunohistochemistry. (**a**) Western blot using the LS-B6098 HK1 antibody detects one band under normal conditions at the appropriate molecular weight in both mouse and rat brain protein extracts. The blot is shown at a normal contrast on the left and higher contrast on the right, which reveals the detection of some lower molecular weight bands. (**b**-**c**) The monoclonal anti-HK1 (LS-B6098) and the rabbit polyclonal anti-HK1 (ab150423) antibodies produce identical signals when used to immunostain primary mouse neurons (**b**) and astrocytes (**c**). Scale bars: 10 μm. (TIFF 1832 kb)

Additional file 4: Figure S3. HK1 immunostaining outside of the SNpc in a 1-year-old WT mouse. (**a**) HK1 immunostaining in the Orexin-A-positive cells that sense glucose in the hypothalamus. Scale bar: 20 μm. (**b**) HK1 immunostaining of the Purkinje cells in the cerebellum shows that the surrounding neuropil contains more HK1 than the Purkinje cell soma. Scale bar: 20 μm. (**c**) HK1-positive astrocytes in the corpus callosum. Scale bar: 50 μm. (**d**) Higher magnification image of HK1 and GFAP immunostaining in an astrocyte located in the corpus callosum. Scale bar: 10 μm. (**e**) HK1 immunoreactivity in an IBA1-positive microglial cell. Scale bar: 5 μm. (TIFF 3321 kb)

Additional file 5: Table S2. Complete list of biochemicals quantified in DJ-1 knockout mouse brains (TIFF 91 kb) (XLSX 21 kb)

Additional file 6: Figure S4. Blood glucose levels in aged DJ-1 knockout mice. Blood glucose measurements taken from the tail blood of 14–15 month old mice that were either fed or fasted for 48 h (*n* = 9 WT fed (5 M, 4 F), *n* = 5 DJ-1 knockout fed (3 M, 2 F), *n* = 9 WT fasted (5 M, 4F), *n* = 5 DJ-1 knockout fasted (3 M, 2 F)). Two-way ANOVA was used to compare the groups. (TIFF 91 kb)

Additional file 7: Table S3. Complete list of mRNAs quantified using RNA-Seq in DJ-1 knockout rat brains. (XLSX 1596 kb)

## Abbreviations

CRYAB: α-crystallin B chain
FDR: False discovery rate
GLUL: Glutamine synthetase
GSK-3β: Glycogen synthase kinase 3β
HK1: Hexokinase 1
HK2VBD: VDAC-binding domain of HK2
iTRAQ: Isbobaric tags for relative and absolute quantification
PD: Parkinson’s disease
PTEN: Phosphatase and tensin homologue
RNA-Seq: RNA-Sequencing
SNpc: Substantia nigra pars compacta
