# Supplementary figures and images for: Hexokinases link DJ-1 to the PINK1/parkin pathway
Source: Mol Neurodegener. 2017 Sep 29;12:70. doi: 10.1186/s13024-017-0212-x (PMC5622528; doi:10.1186/s13024-017-0212-x)

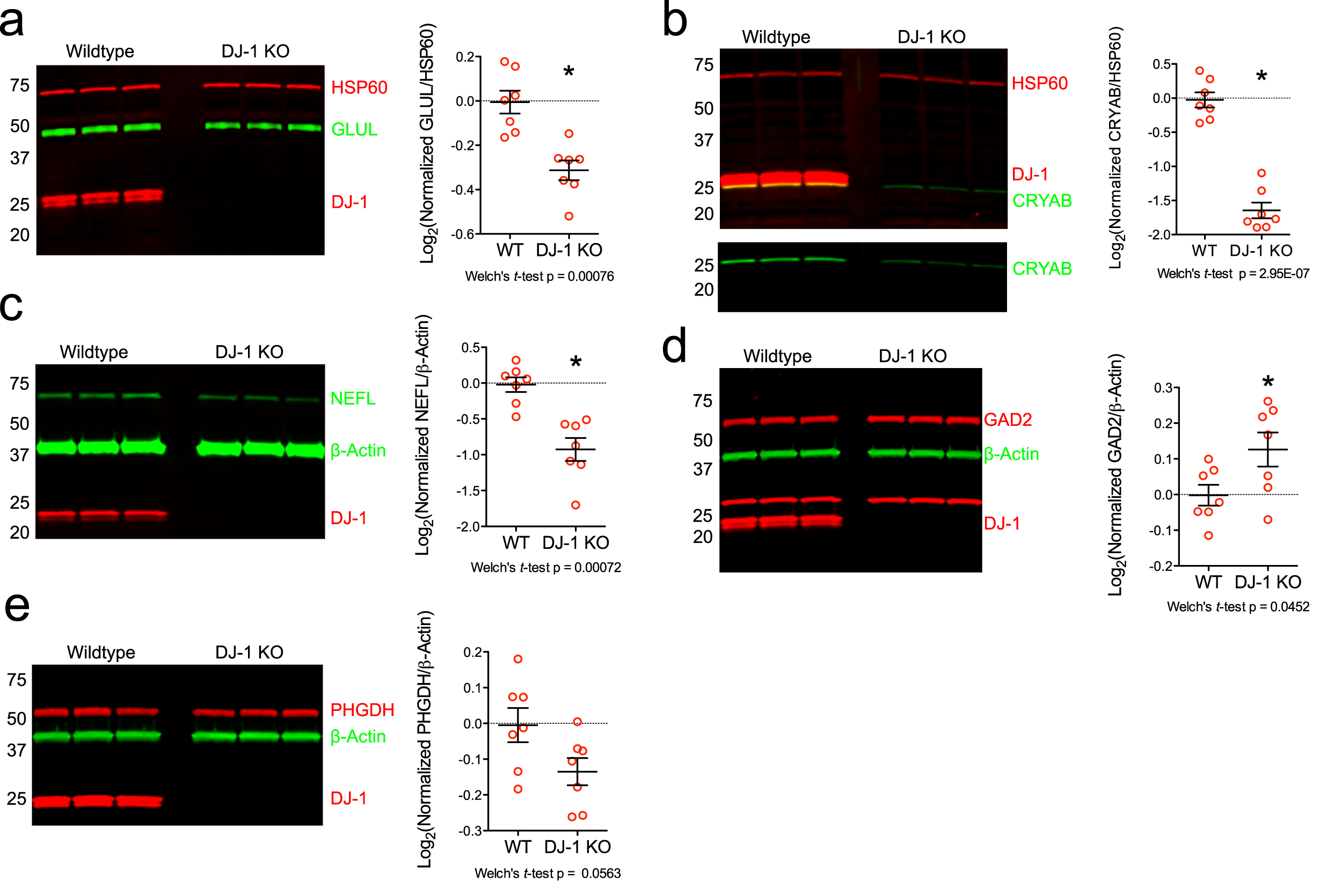

Supplement: Supplementary file 2 — Technical validation of iTRAQ hits in the DJ-1 knockout rat brain by immunoblotting. (a-b) Western blots for GLUL and CRYAB in mitochondria-enriched fractions from 6-month-old DJ-1 knockout rat brains. The mitochondrial protein HSP60 was used as a loading control. Data are graphed as in Fig. 1d. (c-e) Western blots for NEFL, GAD2, and PHGDH in cytosol-enriched fractions from 6-month-old DJ-1 knockout rat brains with β-actin as a loading control. Data are graphed as in Fig. 1d. (TIFF 514 kb) [file 13024_2017_212_MOESM2_ESM.tif]

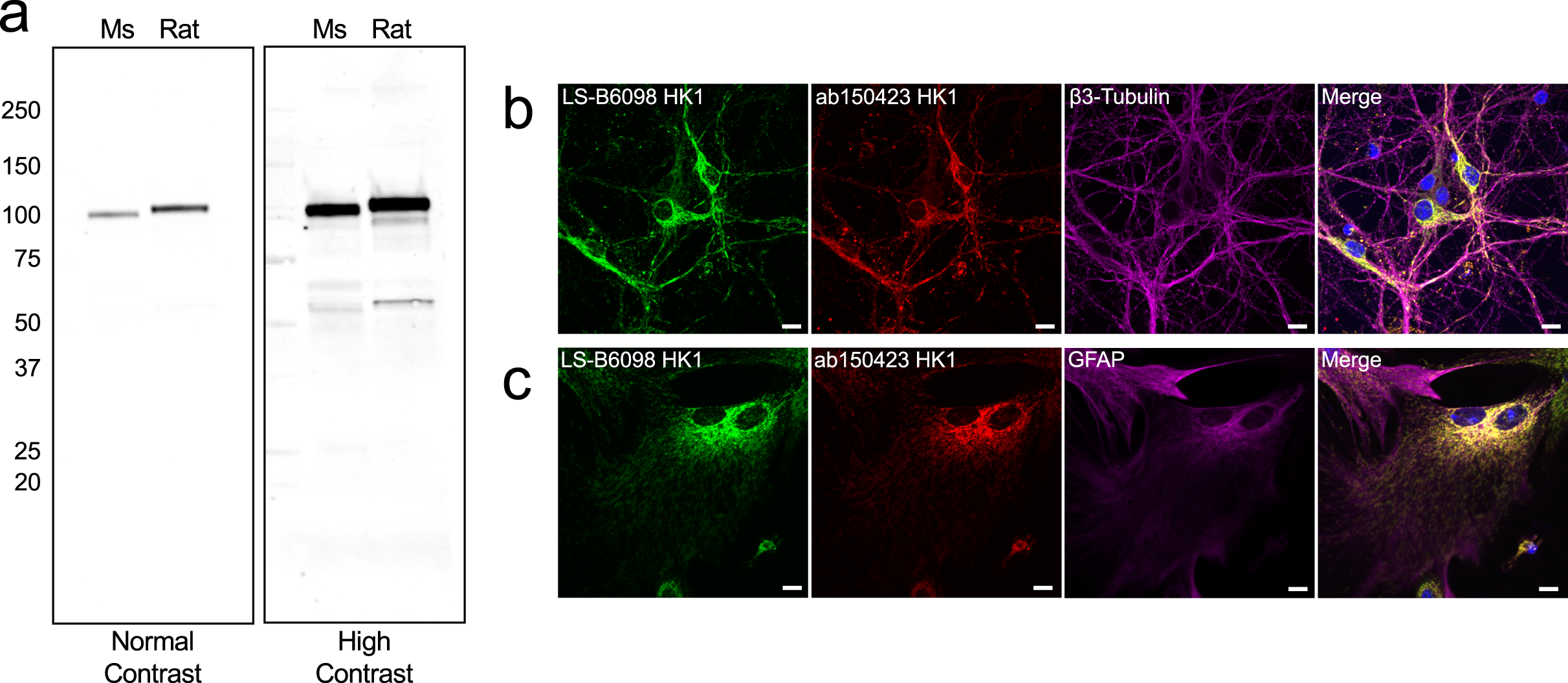

Supplement: Supplementary file 3 — Validation of a monoclonal anti-HK1 antibody for use in immunohistochemistry. (a) Western blot using the LS-B6098 HK1 antibody detects one band under normal conditions at the appropriate molecular weight in both mouse and rat brain protein extracts. The blot is shown at a normal contrast on the left and higher contrast on the right, which reveals the detection of some lower molecular weight bands. (b-c) The monoclonal anti-HK1 (LS-B6098) and the rabbit polyclonal anti-HK1 (ab150423) antibodies produce identical signals when used to immunostain primary mouse neurons (b) and astrocytes (c). Scale bars: 10 μm. (TIFF 1832 kb) [file 13024_2017_212_MOESM3_ESM.tif]

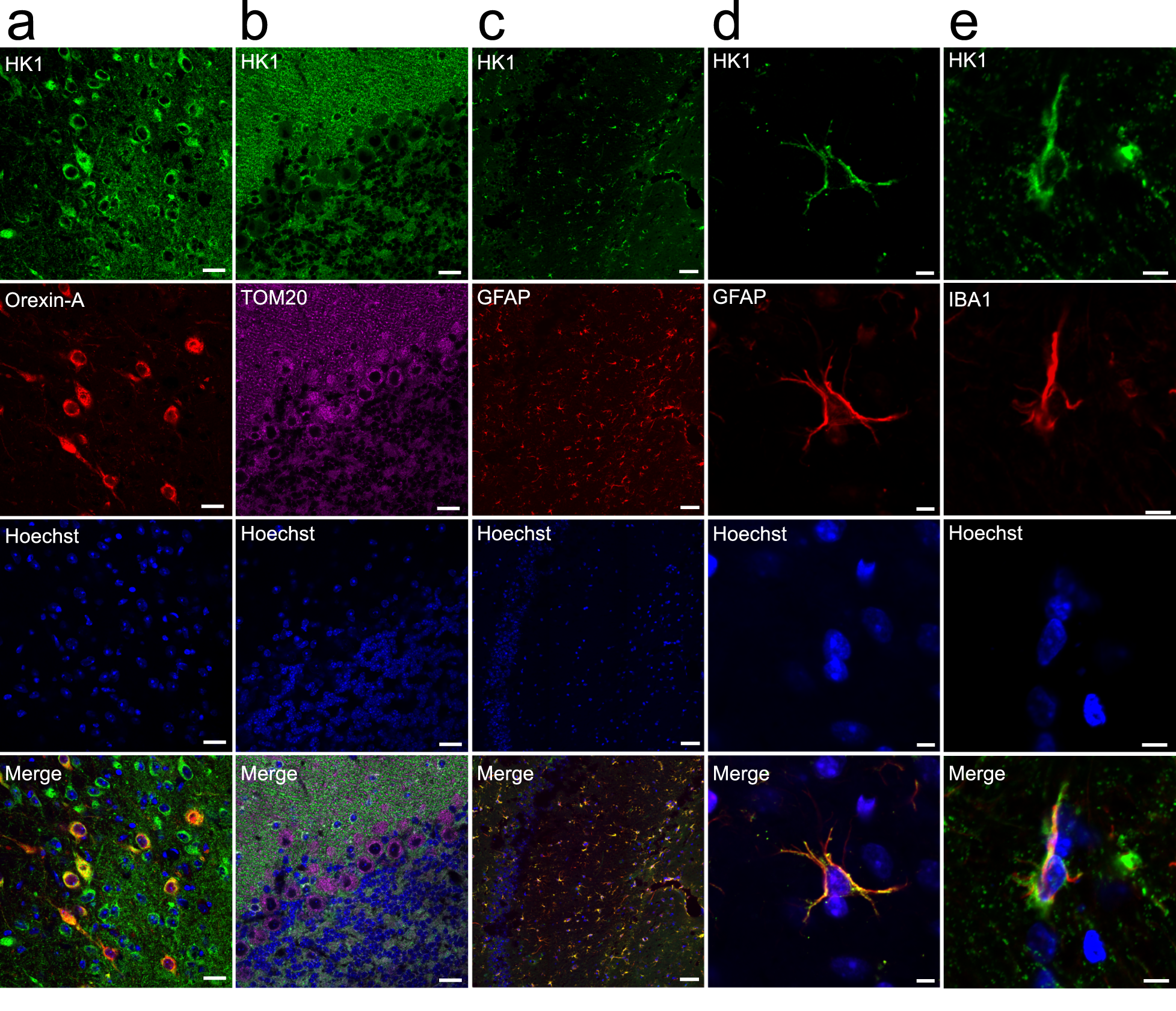

Supplement: Supplementary file 4 — HK1 immunostaining outside of the SNpc in a 1-year-old WT mouse. (a) HK1 immunostaining in the Orexin-A-positive cells that sense glucose in the hypothalamus. Scale bar: 20 μm. (b) HK1 immunostaining of the Purkinje cells in the cerebellum shows that the surrounding neuropil contains more HK1 than the Purkinje cell soma. Scale bar: 20 μm. (c) HK1-positive astrocytes in the corpus callosum. Scale bar: 50 μm. (d) Higher magnification image of HK1 and GFAP immunostaining in an astrocyte located in the corpus callosum. Scale bar: 10 μm. (e) HK1 immunoreactivity in an IBA1-positive microglial cell. Scale bar: 5 μm. (TIFF 3321 kb) [file 13024_2017_212_MOESM4_ESM.tif]

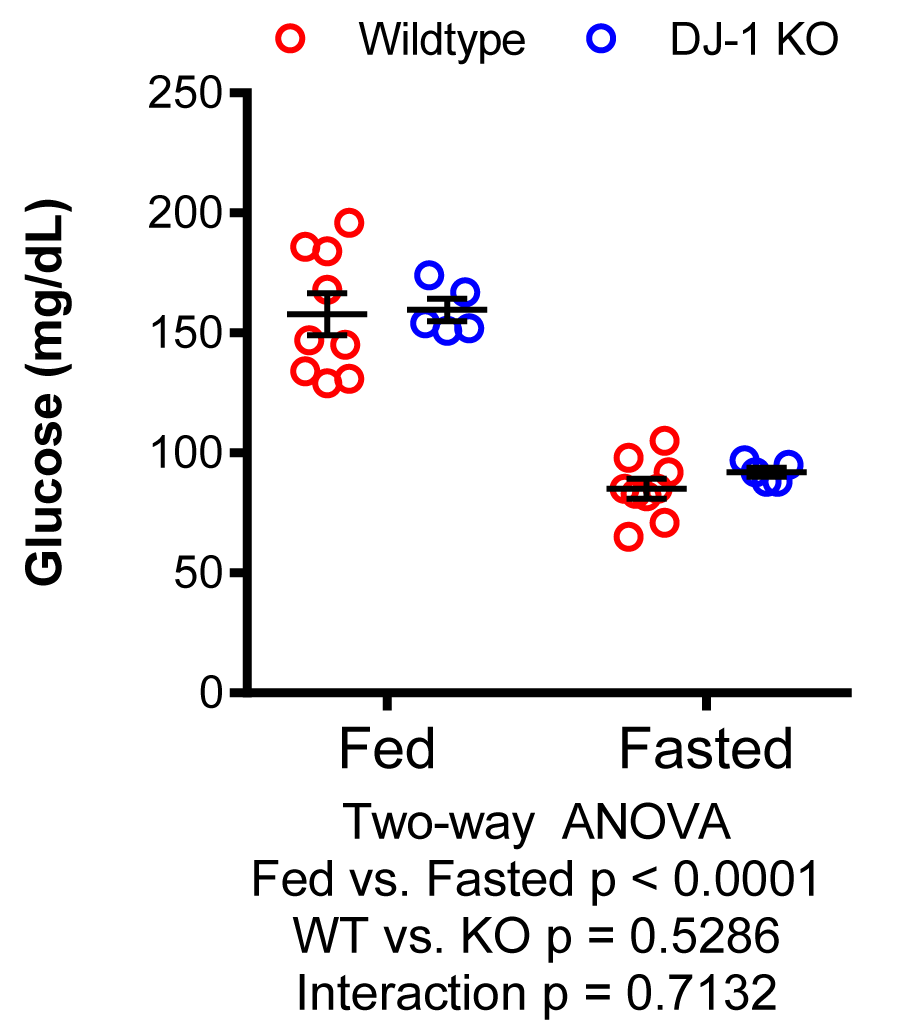

Supplement: Supplementary file 6 — Blood glucose levels in aged DJ-1 knockout mice. Blood glucose measurements taken from the tail blood of 14–15 month old mice that were either fed or fasted for 48 h (n = 9 WT fed (5 M, 4 F), n = 5 DJ-1 knockout fed (3 M, 2 F), n = 9 WT fasted (5 M, 4F), n = 5 DJ-1 knockout fasted (3 M, 2 F)). Two-way ANOVA was used to compare the groups. (TIFF 91 kb) [file 13024_2017_212_MOESM6_ESM.tif]
